# Morphometric Characteristic of Black Soldier Fly (*Hermetia illucens*) · Wuhan Strain and Its Egg Production Improved by Selectively Inbreeding

**DOI:** 10.3390/life12060873

**Published:** 2022-06-10

**Authors:** Minmin Cai, Li Li, Zhengzheng Zhao, Ke Zhang, Fang Li, Chan Yu, Rongfang Yuan, Beihai Zhou, Zhuqing Ren, Ziniu Yu, Jibin Zhang

**Affiliations:** 1 State Key Laboratory of Agricultural Microbiology, National Engineering Research Centre of Microbial Pesticides, College of Life Science and Technology, College of Animal Science & Technology, Huazhong Agricultural University, Wuhan 430070, China; cmm114@mail.hzau.edu.cn (M.C.); zz153837@sina.com (L.L.); zz0606@yeah.net (Z.Z.); zank324@163.com (K.Z.); coreysalinas908@gmail.com (F.L.); renzq@mail.hzau.edu.cn (Z.R.); yz41@mail.hzau.edu.cn (Z.Y.); 2 State Key Laboratory of Biocatalysis and Enzyme Engineering, School of Life Sciences, Hubei University, Wuhan 430062, China; yuchan72@hubu.edu.cn; 3 Beijing Key Laboratory of Resource-Oriented Treatment of Industrial Pollutants, Department of Environmental Science and Engineering, University of Science and Technology Beijing, Beijing 100083, China; yuanrongfang@ustb.edu.cn (R.Y.); zhoubeihai@sina.com (B.Z.)

**Keywords:** black soldier fly, egg production, morphometric characteristic, selective inbreeding, life-history

## Abstract

The use of black soldier fly (BSF) larvae to recycle various organic materials while producing biomass for use as feed is well established. Variety selection is important from the perspective of application. In the current study, morphometric and life-history traits of a Wuhan-domesticated BSF colony (Wuhan strain) were compared to those of a ‘selectively inbred’ population (inbred strain, inbred for 10 generations). In terms of morphological characteristics, the results showed that both strains had dichoptic compound eyes, club-shaped antennae, blue halters, and blue-green metallic luster wings with a hexagon discal cell. In both strains, the body and wing length of female adults were slightly larger than those of male adults. The first four larval stages of the BSF occurred rapidly (1–12 days) with transitions across stages resulting in doubling of size for both populations. Selective inbreeding did not alter the life-history traits of the larval exuviate stage in terms of age, size, weight, and feed reduction rate. Overall egg production for the inbred strain was significantly higher (1.5 times greater) than the Wuhan strain. This is explained by increased adult emergence and individual oviposition performance. It was speculated that inbreeding improved the reproductive success of inbred adult female offspring and selection process steadied it. The findings indicate that selective inbreeding could enhance overall oviposition performance and provide a strategy to selectively breed BSF with high egg production for future applications.

## 1. Introduction

The black soldier fly (BSF; *Hermetia illucens*; Diptera: Stratiomyidae) is a widely distributed insect worldwide. Larvae of the BSF (BSFL) feed on decaying organic matter and are often collected from manure in livestock facilities [1]. Black soldier fly larvae convert these materials to protein, keratin, fat, and so on, which can be processed into high quality feed in agri- and aquaculture; such a process can serve as a sustainable method for waste management [2]. Other advantages of the BSFL are the suppression of pest species, such as the house fly, *Musca domestica* L. (Diptera: Muscidae) [3]; reduction of pathogens [4,5], odors [6], and antibiotics [7]; and concerns with antibiotic-resistance in the associated microbial communities [8]. Furthermore, the fat from BSFL can be used to create bio-energy as well as antimicrobial peptides [9,10].

The impact of BSF inbreeding has not previously been examined. Zhou et al. [11] suggested that BSFL of a Wuhan-domesticated strain are a good choice for waste management due to conversion rate advantages compared with two other colonies in Guangzhou, China, and Texas, USA. For effective utilization, studying the impact of inbreeding on BSF production is critical. The objectives of the current study were to compare morphometrics and life-history traits for a strain that has been maintained long-term (i.e., the Wuhan strain) to an ‘inbred’ strain originating from a single egg clutch collected from the Wuhan strain. Data from such experiments could shed light on the overall efficiency and productivity of the BSF, resulting in recommendations for optimizing the system either through isolation or periodic insertions of new genetic material.

## 2. Material and Methods

### 2.1. BSF

**Figure 1:**
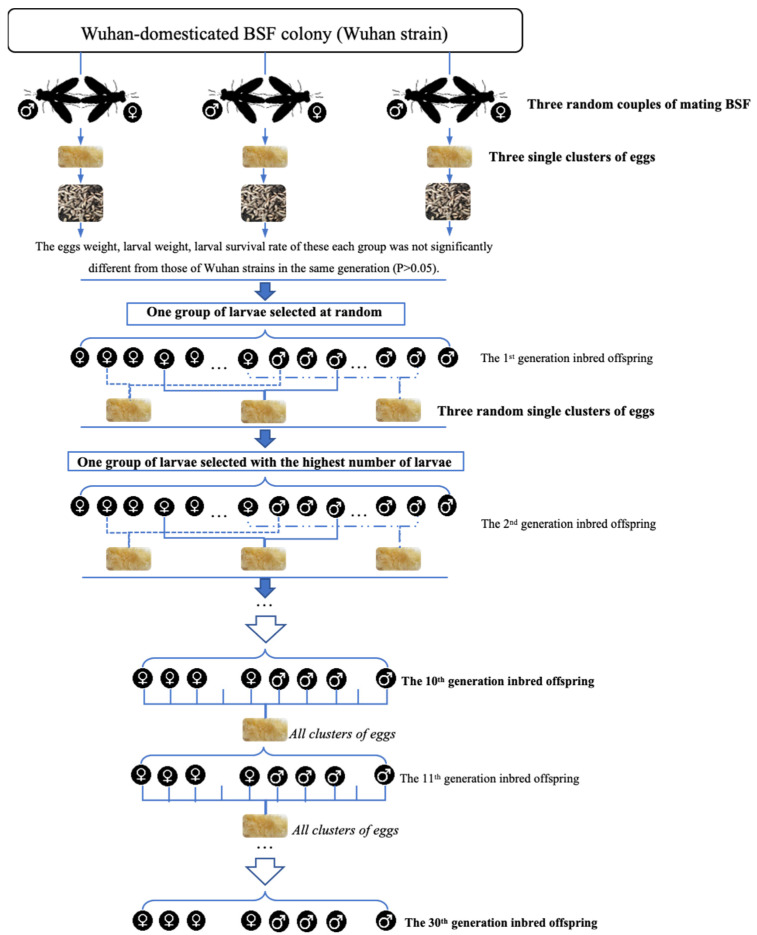
Selective inbreeding process of the “inbred strain”.

The Wuhan BSF strain (Wuhan strain) was established from eggs collected at a poultry facility in Wuhan, China in 2011. The ‘inbred’ strain came from one cluster of eggs from a single female BSF of the Wuhan strain, whose egg weight, larval weight, and larval survival rate were not significantly different from those of Wuhan strains in the same generation. These eggs were selectively inbred for 10 generations. Each generation of the inbred strain was propagated from a single selected egg clutch (with the most hatched larvae) from the previous generation (Figure 1 provides details of the selective breeding program).

Due to inbreeding depression (less mating and survival) of the 10th generation inbred BSF, the following generation was propagated from all clusters of the previous generation. This was carried out for 20 more generations to eliminate the inbreeding depression and increase the inbred population.

### 2.2. Colony Methods

The Wuhan and inbred BSF colonies were located in a greenhouse, where the temperature was controlled to be within the range of 27 °C to 30 °C, with a relative humidity of 60–70%. Larvae from both strains were fed wheat bran and wheat flour at a ratio of 6:4 (*w*:*w*, final water content: 60–70%), on the basis of the standard scheme established by Sheppard et al. [12]. The feed residues were cleaned, and new food was added every three days until 50% of the BSFL reached the prepupal stage, in which the color of larvae turned black-gray. The larval and prepupal stages lasted between 20–24 days.

**Figure 2:**
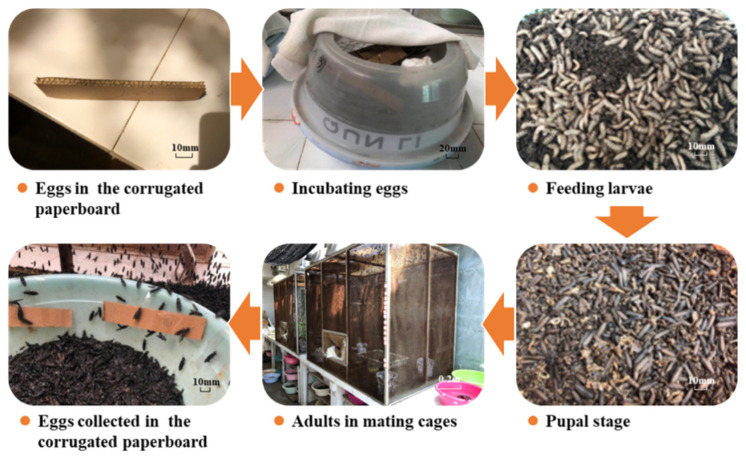
Colony process of BSF (Black solider fly).

After all larvae become prepupal, they were separated from the residues and put into a dry barrel in the dark for pupation. The pupae were moved into a mating cage with an LED light (50 watts; ~3500 Lux of illumination in the middle of cage), which was lit from 7:00 to 17:00 every day following pupa eclosion. In the mating cage, the adults were mating and spawning. The eggs were collected from corrugated paperboards, which were positioned above the fermented artificial feed (wheat bran and wheat flour at a ratio of 6:4 *w*:*w*, final water content >90%; naturally fermented for 3–7 days at room temperature) with a sour odor to attract adults for spawning. The collected eggs were incubated at 28 °C and 60–70% relative humidity in a dark place to avoid sunlight and water evaporation. The whole colony process lasted between 38–45 days for one generation (Figure 2).

### 2.3. Morphological Characteristics of Adults

Morphometrics of BSF were based on key taxonomic characteristics of the adults [13,14]. These characteristics spanned the morphology of head (eye and antenna), thorax (prothorax, mesothorax, and metathorax), leg, wing, halter, abdomen, and genitalia. A stereomicroscope (Wuhan Ruitu Technology Co., Ltd., Wuhan, China) was used to measure these traits.

### 2.4. Growth Characteristics of BSFL

Approximately 300 6-day-old Wuhan and inbred strain BSFL were placed in a plastic container (diameter, 32 cm; height, 13.5 cm) and fed with artificial feed (560 g artificial feed; dry weight, 197.5 g). Ten larvae were randomly collected every two days, and their size (body length and width) was measured after cleaning. Then, the larvae were returned into the container.

The larvae in the prepupal stage were separated from the residue when 50% of them had turned black. The water content of the substrate (10 g) was determined by collecting and drying to constant weight at 60 °C. The feed reduction rate was calculated using Equation (1). All experiments were carried out in triplicate.

Feed reduction rate = (dry weight of original feed − dry weight of residues)/(dry weight of original feed) × 100%(1)

### 2.5. Oviposition Characteristics of Adult BSF

In the greenhouse previously described, (28 °C, 60–70% humidity, LED lit from 7:00 to 17:00 every day), 250 pupae (in a small container) of the Wuhan and inbred strains were placed into separate adult-mating cages (*n* = 3). The volume of the cage was approximately 0.025 m^3^ (0.05 m^2^ × 50 cm). The eggs were collected from honeycomb cardboard every day (placed on the fermented feed at 8:00 a.m. and collected at 4:00 p.m.) and weighed to check the hatch percentage of each strain.

The above mating experiment was repeated four times due to the significant difference in the spawning characteristics between the two strains (*n* = 9). The eclosion rates, female–male ratios, and weights of eggs laid by one female adult were calculated using Equations (2)–(4), respectively.

Eclosion rate = eclosion adult number/pupae number × 100%(2)

Female–male ratio = female adult number/male adult number(3)

Egg weight per one female = total collected egg weight/female adult number(4)

### 2.6. Statistical Analysis

The extent of differences between the two strains was gauged using Student’s *t*-tests executed in Microsoft Excel (Bill Gates, Redmond, WA, USA, version 16.16.18); α = 0.05 was used as the benchmark for statistical significance.

## 3. Results

### 3.1. Morphological Characteristics of Adult BSF

Morphological characteristics of adult BSF from the two strains under the same breeding conditions are summarized in Table 1. The body and wing of male adults of the Wuhan strain (13.0–17.0 and 10.0–13.0 mm, respectively) are longer than those of the inbred strain (12.0–16.5 and 9.0–12.0 mm, respectively), although these differences are not statistically significant (*p* > 0.05). For both strains, the body and wing lengths of female adults are slightly greater than those of male adults for both strains.

**Figure 3:**
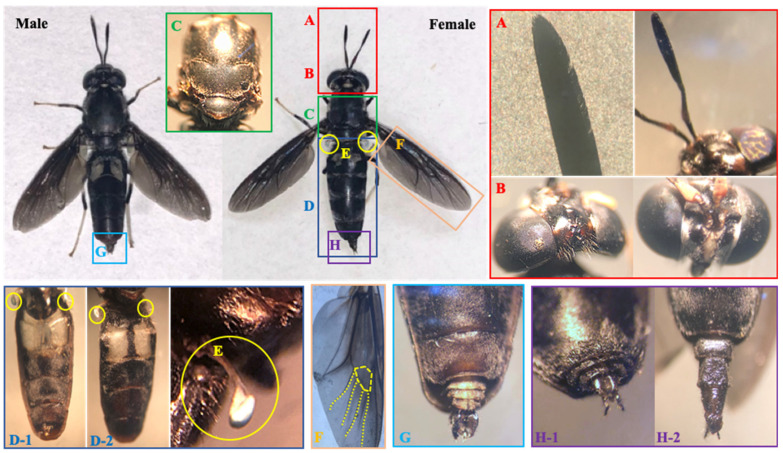
Morphological characteristics of adult BSF, Wuhan strain: (**A**) antenna and its last segment (flagellum); (**B**) head; (**C**) mesotum (thorax); (**D-1**) abdomen; (**D-2**) abdomen in dorsal view; (**E**) halter; (**F**) wing; (**G**) male genitalia; (**H-1**) contracting female ovipositor; and (**H-2**) porrect female ovipositor.

The external morphological structures of female and male adult BSF are shown in Figure 3. The head of the adult BSF (Figure 3B) is similar in width to the thorax. The ocelli distribution on the head apex is triangular (named ocellar triangle). Their compound eyes occupy most of the head and are dichoptic for male and female adults. The mouth parts are black and covered with hair. Two antennae are close to each other (Figure 3A). The segments of the antennae are club-shaped and consist of scape (proximal antennomere, black), pedicel (second antennomere, yellow), and flagellum (a flat terminal segment, black). The flagellum is 4–5 times longer than the pedicel segment. The length of the antenna is important for sensory perception, and some of the sensory uses of antennae include motion, orientation, odor, sound, humidity, and a variety of chemical cues [15].

The thorax consists of the prothorax, mesothorax, and metathorax. The mesothorax (Figure 3C) is well-developed and relatively large; it is black, covered with dense short setae, and includes the mesotum, transverse suture, and scutellum. Spines occur on the sides of seutellum. Two blue halters (Figure 3E) are located on the sides of the metathorax and have a white transparent handle, which may maintain balance for flying. The thorax bears the only pair of wings with internal flight muscles and is an important base for adults flying with wings [15]. The wings (Figure 3F) show blue-green metallic luster under sunlight with a discal cell (hexagon). Four medial veins emit from the discal cell and gradually disappear near the wing edge. This hexagon discal cell and veins may help the wings maintain structural stability and level when flying.

The adults have six legs, composed of coxa, trochanter, femur, tibia, and tarsus for each leg. The tarsus is white, and the others are black with short setae. The abdomen is long and slender, covered with dense short hairs, and has five visible segments (Figure 3D). They may protect the insect’s internal anatomy from the outside environment. Two hairless translucent windows are located on the first and second visible segments. The windows look blue-green at the early stage of adulthood, change to white, and become colorless and empty at the later period to death. These segments are speculated to store energy (i.e., fat), which is completely consumed by adults for living, mating, breeding, and death, or possible bioluminescent signaling [15].

Male genitalia, which consists of aedeagus (center) and parameres (sides), are short and exposed to the environment (Figure 3G). The female terminalia has three segments, and the distal segment is the ovipositor. These segments can be invaginated and are flexible, which keeps the ovipositor in the body normal and enables stretching for the purpose of laying eggs into a suitable gap (Figure 3H).

These morphological characteristics of adults conform with those of *H. illucens* L., according to the taxonomic key list [14], for Stratiomyidae, Diptera, including subfamilies, genera, and species of Chinese Stratiomyidae. The inbreeding process did not change them in the current study.

### 3.2. Exuviate and Age Characteristics of BSFL

Observing the exuviate by using a stereomicroscope reveals that the BSFL pass through six instars with five exuviate stages. The exuviate and growth characteristics of BSFL are shown in Table 2.

**Figure 4:**
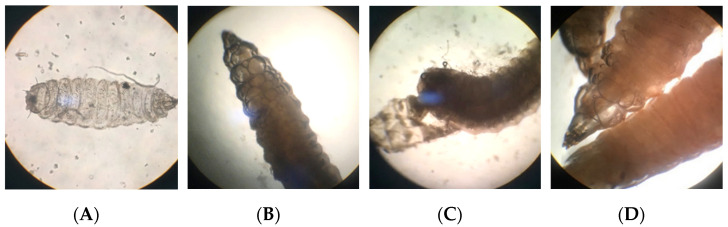
Different ecdysis stages of BSFL. (**A**) 1st exuviate; (**B**) early stage of 2nd exuviate; (**C**) late stage of 2nd exuviate; (**D**) 3rd ecdysis.

The first exuviate of larvae occurs 1–2 days after hatching, in which the body length of the larva is only 1.5–2.0 mm. The shedding skin is semipermeable (Figure 4A). Normally, the 1st instar of BSFL starts from hatching to this exuviate.

The 4–5-day old larvae entered into the second exuviate period, and the body length was twice that of the 1st instar. When the exuviate started, many bubbles separated the old skin and the body (Figure 4B). The larvae broke the tail of old skin and retreated (Figure 4C) because the larval tail was wider than the forepart.

The larva was macroscopic from the 3rd exuviate, when larval length reaches about 8 mm, growing for 6–7 days (Figure 4D). The 4th and 5th exuviate stages occurred in 11–12- and 18–20-day-old BSF. After the 5th exuviate, the 6th instar of BSFL, which are named as prepupa, turned dark gray.

### 3.3. Growth of BSFL

**Figure 5:**
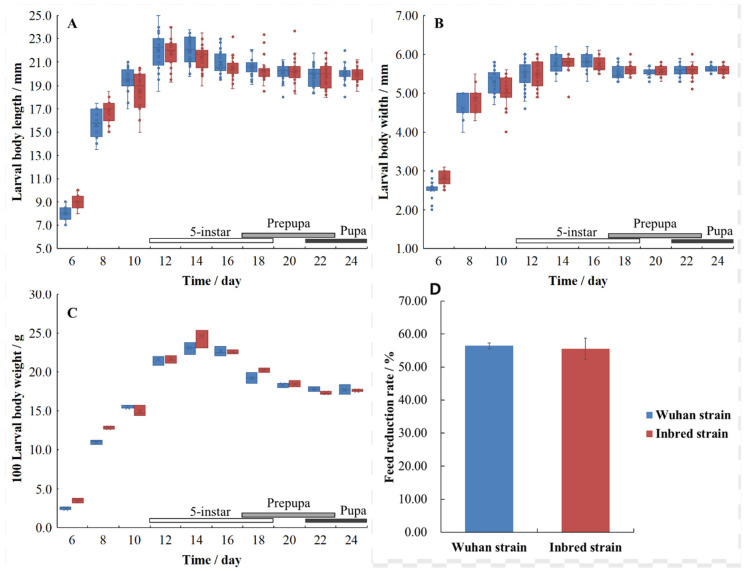
Body length ((**A**) *n* = 30), body width ((**B**) *n* = 30), body weight ((**C**) *n* = 3), and feed reduction rate ((**D**) *n* = 3) of the two BSFL strains in the growing process.

The growth parameters of the two BSFL strains are shown in Figure 5. The growth parameters of the inbred strain are not significantly different from those of the Wuhan strain. The body length, width, and weight of both strains increased rapidly from the 6th day to the 12th day but decreased slightly after the 14th day. They reached maximum values (22.0 ± 1.2 mm × 5.76 ± 0.22 mm, 23.1 ± 0.61 g/100 larvae for the Wuhan strain; 21.4 ± 1.0 mm × 5.78 ± 0.21 mm, 24.6 ± 1.08 g/100 larvae for the inbred strain; 14th day) when BSFL grew to the 5th instar stage and decreased in the prepupa and the pupa stages. Overall, their size and weight followed the order: 5th instar > prepupa (6th instar) > pupa. The feed reduction rates of the Wuhan (56.4 ± 0.9%) and inbred (55.5 ± 3.2%) strain are not significantly different.

### 3.4. Difference in Oviposition Performance

**Figure 6:**
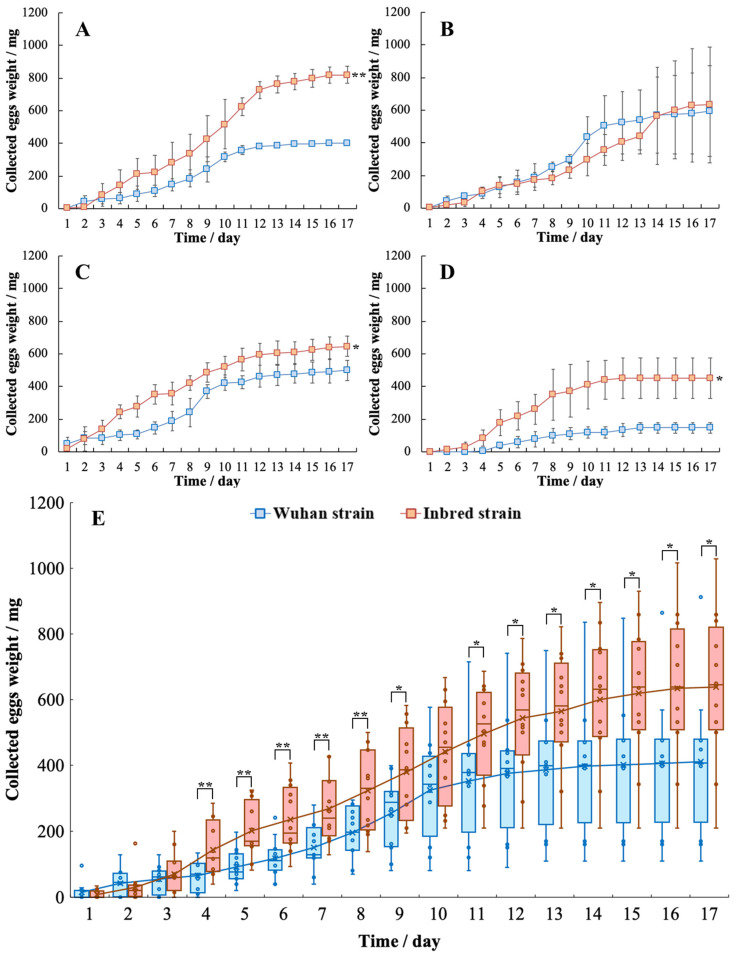
Collected egg weight of the two BSF strains in spawning systems containing 250 pupae (**A**–**D**), four independent repeated trials, *n* = 3; (**E**) statistical analysis of all data, *n* = 12; *, 0.01 < *p* < 0.05; **, *p* < 0.01).

Four individual repeated trials were performed to test the difference in oviposition performance between the Wuhan and inbred strain, and results are shown in Figure 6A–D. The student’s *t*-test was used to evaluate significant differences for each individual trial and the paired-samples *t*-test for the all data (Figure 6E). The BSF oviposition for both strains can last for 8–17 days, whereas that of females collected in Tsukuba, Japan is 10–12 days [16]. A significant difference in gained eggs was observed between the Wuhan and inbred strain in trials A, C, and D. The final egg weight of the inbred strain (637 ± 227 mg [49,049 ± 17,479 eggs]) was significantly heavier by 1.55 times than that of the Wuhan strain (411 ± 213 mg (31,647 ± 16,401 eggs); Figure 6E) in systems containing 250 pupae.

**Figure 7:**
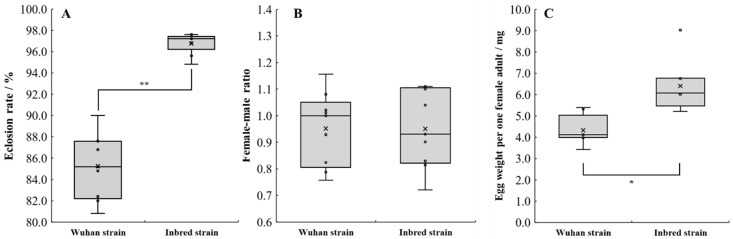
Eclosion rate (**A**), female–male ratio (**B**), and egg weight laid by one female adult (**C**) of the two BSF strains (*n* = 9; *, 0.01 < *p* < 0.05; **, *p* < 0.01).

The eclosion rate, female–male ratio, and egg weight laid by one female are calculated to understand the reason for the oviposition advantage of the inbred strain, and results are shown in Figure 7. The female–male ratios of the Wuhan and inbred strain are 0.95 ± 0.14 and 0.95 ± 0.15, respectively, and the difference is not statistically significant. However, the other two parameters display a significant difference. The eclosion rate and the egg weight laid by one female of the inbred strain (96.8 ± 0.9% and 6.4 ± 1.2 mg or 492 ± 92 eggs, respectively) are significantly higher than those of the Wuhan strain (85.2 ± 3.1% and 4.3 ± 0.7 mg or 331 ± 54 eggs, respectively).

## 4. Discussion

### 4.1. External Morphological Characteristics of Adult BSF

The adult sizes of both strains, i.e., Wuhan and inbred strain, are not significantly different. The detected body length (12.0–18.0 mm) in this study was longer than those where BSF were fed by meat meal (8.06–9.79 mm) and similar to those fed by hen feed (15.47–16.22 mm) [17]. The larva diet can affect the size of adult BSF. This finding means that the artificial feed with wheat bran and flour used in this study can provide adequate nutrition for BSF growth, comparable to hen feed, and is beneficial for their breeding.

The body and wing lengths of female adults are slightly larger than those of male adults for both strains, and these results are consistent with those of a previous study [17]. This may be because female bodies need to prepare for future spawning.

The details of external morphological structure of female and male adult BSF for both strains are characterized and show no statistically significant difference. All external morphological structures conform to the characteristics of *H. illucens* (Diptera: Stratiomyidae) [13,14].

### 4.2. Growth Characteristics of BSFL

The BSFL grow through six instars with five exuviate stages, and this phenomenon is consistent with previous research [18]. Results show that the first four exuviate stages of the BSFL occur at the rapid growth stage (1–12 days), and each exuviate tends to be associated with a doubling of body size. The last exuviate stage marks the transition from larva to prepupa, with ceasing of eating for further pupating (color: black). The BSFL development time, which also affects the exuviate time, is influenced by diet, temperature, toxic compounds, and water content [19,20,21,22].

The larval size and weight reach their maximum (21.4 ± 1.0–22.0 ± 1.2 mm and 23.1 ± 0.61–24.6 ± 1.08 g/100 larvae, respectively) when they grow to the 5th instar stage for both strains. By contrast, the BSFL from a private company (CIMI S.r.l., Cervasca [CN], Italy) achieved maximum length and weight of approximately 18 mm and 18 g/100 larvae, respectively, for vegetable and fruit waste rearing [23]. The BSFL strain maintained at the Laboratory of Entomology, Wageningen University (Wageningen, the Netherlands) had an average fresh weight of 6.8–8.3 g/100 larvae when fed with chicken, pig, and cow manure [24]. The larvae in this study were relatively big and heavy, which may be beneficial for increased larval harvest yield and easy separation from substances for further application. It is recommended that the 5th instar BSFL be harvested to obtain the maximum yield. However, the prepupa of BSFL is an alternative for harvesting due to the self-harvest by redirecting their natural search for pupation sites into collection bins [1,25] and emptying the intestine for pupa prepared.

In terms of feed reduction, both strains can reduce 56.4 ± 0.9%–55.5 ± 3.2% of dry feed mass. In previous studies, BSFL of the Wuhan strain have been fed with dairy manure and soybean curd residue, mushroom root waste, and chicken manure with 26–72% [26], 42.3% [26,27], and 35.8–40.5% [28], respectively, of reduction. The feed reduction rates are affected by the feed type and can be improved by decreasing the feed mass. For example, 300 BSFL fed with 27 and 70 g dairy manure daily reduces the manure dry matter mass by 58% and 33%, respectively [29]. Therefore, the ratio of feed to larvae can be controlled to improve the feed reduction for further application.

### 4.3. Oviposition Performance Difference for Both Strains

A significant difference in gained eggs is observed between the two strains, and the inbred strain displays better oviposition performance than the Wuhan strain. The final gained egg weights of the Wuhan and inbred strains are 411 ± 213 mg (31,647 ± 16,401 eggs) and 637 ± 227 mg (49,049 ± 17,479 eggs), respectively, in the systems containing 250 pupae. Light sources are reportedly a key factor for fertilizing eggs [16,30]. In this study, an LED light was used for 10 h every day to provide a stable light source for all systems. By contrast, in the BSF obtained from *Hermetia* Baruth GmbH (Baruth, Germany), the total egg weight was 64.8–68.9 mg in cages containing 80 adults (40 males and 40 females) under LED light (202–215 mg per 250 adults) [31]. Nakamura et al. [16] collected 289.0 ± 27.0 eggs per female (from Tsukuba, Japan) under LED light (36 125 ± 3375 eggs per 250 adults). Thus, under the same light sources, the oviposition performance of the inbred strain is better than that of the Wuhan strain.

This beneficial oviposition performance of the inbred strain is due to a high eclosion rate (96.8 ± 0.9%) and egg weight laid per female adult (6.4 ± 1.2 mg/female or 492 ± 92 eggs/female) of the inbred strain. The egg weights per female of the BSF from Baruth, Germany and Tsukuba, Japan are 1.6–1.7 mg and 289.0 ± 27.0 eggs, respectively [16,31]. Our inbred strain therefore has a significant reproductive advantage compared to the Wuhan strain and others.

The mechanism of the reproductive advantage for the inbred strain is still unclear. Inbreeding causes inbreeding depression in survival, mating, reproduction, and fitness and leads to an increase in genome-wide homozygosity and increased expression of deleterious recessives [32,33]. The inbreeding depression for the 10th generation inbred BSF is observed due to less mating and lower survival of the offspring to adulthood, despite the selection with the highest number of larvae in the three random single clusters of eggs for each generation. However, after the collection of all eggs from the 10th generation inbred BSF and normally breeding them for more than 20 generations, the inbreeding depression is mitigated and BSF displays the reproductive advantage.

Studies have revealed that inbreeding does not influence fertility or productivity in *Drosophila* and flour moths (*Ephestia kühniclla* Z.) [34,35]. Furthermore, Meunier and Kölliker [36] demonstrate that sib mating (leading to inbred offspring) does not influence the reproductive success of European earwig (*Forficula auricularia*) parents and reveals that inbreeding dramatically depresses the reproductive success of inbred adult male offspring but only has little effect on the reproductive success of inbred adult female offspring. The current results suggest that the inbreeding of BSF may also have little effect on the reproductive success of inbred adult female offspring but improves the egg production, which may be a self-strategy to prevent population inbreeding depression at the macro level. Moreover, the selection process may steady the traits of reproductive advantage—more egg production.

For the genetic mechanisms, the inbreeding depression is because part of the genetic load of populations, known as inbreeding load, is only expressed in homozygotes [37]. The inbreeding-enhanced egg production may be because of part of the genetic load. The recessive genes related to oviposition in BSF are inhibited in heterozygotes and expressed in the homozygotes. Thus, the reproductive advantage of the inbred strain is selected by the current selective inbreeding process and kept in the following breeding process. However, the above hypothesis needs further experiments to demonstrate the genetic mechanism of the improved reproductive performance of the inbred strain on the basis of genomic sequencing and analysis of BSF [38].

## 5. Conclusions

In conclusion, the external morphological structure characteristics of the Wuhan and inbred strain, which are identical and conform to the characteristics of *H. illucens* (Diptera: Stratiomyidae), are observed. The larval growth characteristics show that both strains have size and weight advantages. Furthermore, the inbred strain displays a higher reproductive advantage than the Wuhan strain and other strains in previous studies. This may reflect inbreeding selection, which could improve the egg production of inbred BSF adult female offspring. The results therefore provide a basis for one BSF inbreeding selection strategy which could be used in future applications.

## Figures and Tables

**Table 1:** Body and wing lengths of adults from the two BSF strains.

	Wuhan Strain		Inbred Strain	
	Male Adult	Female Adult	Male Adult	Female Adult
**Body length/mm**	13.0–17.0	13.8–18.0	12.0–16.5	13.5–18.0
**Wing length/mm**	10.0–13.0	10.2–14.0	9.0–12.0	10.0–13.5

**Table 2:** Exuviate and growth characteristics of BSFL.

Exuviate Order	Age/Day	Body Length/mm
1	1–2	1.5–2.0
2	4–5	4.0–5.0
3	6–7	~8.0
4	11–12	~15.0
5	18–20	>20.0

## Data Availability

No applicable.
